# Sharenting: characteristics and awareness of parents publishing sensitive content of their children on online platforms

**DOI:** 10.1186/s13052-024-01704-y

**Published:** 2024-07-30

**Authors:** Maria Giulia Conti, Fabiola Del Parco, Francesca Maria Pulcinelli, Enrica Mancino, Laura Petrarca, Raffaella Nenna, Greta Di Mattia, Luigi Matera, Domenico Paolo La Regina, Enea Bonci, Cinthia Caruso, Fabio Midulla

**Affiliations:** 1 Department of Maternal Child and Urological Sciences, Sapienza University of Rome, Rome, Italy; 2 The Italian Pediatric Society, Rome, Italy; 3 Umberto I Teaching Hospital, Viale Regina Elena 324, Rome, 00161 Italy

**Keywords:** Sharenting, Social media, Children privacy

## Abstract

**Background:**

The term “sharenting” describes the increasingly popular habit of parents to share photos, videos, or other information regarding their children on their social profiles, through online platforms. It is highly likely that many parents are posting content about their underage children online with little knowledge of the risks associated with this practice. This study aims to investigate whether variables such as parents’ age, gender, marital status, occupation and educational level influence the practice of sharing child-related content and the degree of awareness.

**Methods:**

We performed a pilot cross-sectional study, based on an anonymous questionnaire. The questionnaire was administered to parents of underage children attending the pediatric outpatient clinic of the Umberto I Hospital, Sapienza University, in Rome, Italy, by researchers, through the google forms platform; qualitative variables were generated on excel sheets and a statistical analysis was performed on SPSS Ibm-statistics using the chi-square test.

**Results:**

Two hundred twenty-eight parents of children under 18 years of age completed the questionnaire (82% mothers, 18% fathers); 98% of the respondents used social media and 75% of them published their children’s related content online. Thirty-one percent of the compilers started their practice of sharenting in the first 6 months of life of their child. Our analysis showed that compared to parents who do not post online, parents who usually post online their children are significantly more likely to be partial employees or unemployed (*p* = 0,002), with lower educational level (*p* = 0,05), younger (less than 35 years of age (*p* = 0,01)) and have a higher number of followers (*p* < 0,001). Finally, 93% of the compilers were not aware of the current legislation and of the risks related to the practice of sharenting.

**Conclusions:**

Pediatricians, healthcare assistants and preventive healthcare professionals should play a central role in alerting parents and families to the risks of sharenting; the results of our study could draw their attention to the increasing practice of sharenting and make healthcare professionals active part in the protection of children.

## Background

The term “sharenting,” is the combination of “sharing” and “parenting,” and describes the increasingly popular habit of parents to share photos, videos, or other information regarding their children on their social profiles, through online platforms [1]. The social media most frequently used for this practice are Facebook (54%), Instagram (16%) and X (former Twitter) (12%) [1]. The risks and consequences of sharenting are varied in nature and complex to fully comprehend, as they also involve legal issues related to child image protection, data privacy and digital security.

This growing phenomenon can expose children to several risks, including identity theft, sexual exploitation, and future emotional distress related to sharing emotions and moods.

As soon as images or videos are posted online, any effective control over them is lost, ownership of photos uploaded by parents to social media is lost and it is generally difficult to obtain their removal due to a substantial lack of legislation [2]. According to a recent investigation by the Wall Street Journal and researchers of the Stanford University and the University of Massachusetts Amherst, Instagram’s algorithms actively promote pedophile networks that commission and sell child pornography content from the popular Meta4 image-sharing app [3].

Although sharenting is often an unintentional and unaware habit among young parents, a new sensitivity about this phenomenon has been reported. A recent survey of 427 people in Turkey found that 86.9 percent of participants said that the sharing of children’s photos and videos on social media platforms by parents, relatives and caregivers can be assessed as child neglect and abuse [4]. Some parents decide to practice conscious sharenting by implementing different strategies (i.e., covering or blurring the face and cutting recognizable parts from the photo) to balance social pressure to constantly post updates about their family and children’s habits and their desire to protect their child’s privacy [5].

Another risk-related phenomenon is the use of online gaming apps to entertain minor children.

A report by the Cybercrime Analysis Unit—UACI—of the Italian State Police, which deals with various types of online child abuse, cyberbullying and child pornography, shofws that younger children, within the age of 10, are often lured into online platforms, linked to free online gaming apps, accessible through their parents’ smartphones [6].

Although the practice of “sharenting” is widespread, especially in Western countries, the reasons and the psychological mechanisms behind sharenting are not fully described as the current literature mainly focuses on the risks and benefits and on the ethical and privacy concerns related to this practice [7]. This study aimed to describe the socio-cultural characteristics of parents practicing sharenting, the reasons and awareness of the potential risks related to this practice. We have also investigated the same features of parents which allows their children to use online gaming apps.

## Methods

We conducted a pilot cross-sectional study, based on an anonymous multiple-choice questionnaire of 27 questions between January and April 2024 at Umberto I Teaching Hospital. After informed written consent was obtained, a researcher submitted the questionnaire to the parents while they were waiting before the pediatric outpatient visit through a link by framing a specially generated QR code using the Google forms application. A literature search was conducted, and the questionnaire form was prepared by the researchers based on a previous recently published survey [4]. The survey was anonymous and was arranged in compliance with the Data Protection Authority. The average duration for completing the questionnaire was about 2 min.

Variables such as child’s age, nationality, age range of both parents, marital status, level of education and length of employment of both parents were investigated through eleven questions.

Occupational status was defined as “Not employed” or “not fully employed” if unemployed, partially employed, or in parental leave. Educational level was defined as lower (primary and secondary school diploma or none) or higher (diploma, degree, PhD).The parent compiler was asked about his/her habit to use social platforms and which; if he/her posts online content about his/her children; how often and which type of content; the possible reasons; what was the level of satisfaction after they received “likes” on the content. We also asked if the parent let his/her children use online game apps. Finally, six questions were asked about the conscious use of social media to assess the level of awareness of these parents, including questions about the knowledge of related legislation, related risks (sensitive data theft, permanents of online content etc., see Tables 1 and 2 legends). The last question investigated the feeling (anxiety, interest, indifference, annoyance) generated by completing the aforementioned questionnaire.

**Table 1:** Analysis of socio-cultural characteristics between parents who publish (G p) child content and parents who do not publish (G np)

Variables	G p	G np	*P* value
Partial employment	90/170 (53%)	17/58 (29.3%)	**0.002**
Lower educational status^a^	21/170 (12.4%)	2/58 (3.4%)	**0.05**
Not aware^b^	160/170 (94.1%)	53/58 (91.3%)	0.4
Single parent	28/170 (16.4%)	6/58 (10.3%)	0.2
Parent age < 35y	60/170 (35.3%)	10/58 (17.2%)	**0.01**
Child age < 3y	65/170 (38.2%)	21/58 (36.2%)	0.7
N° followers > 500	36/170 (21.2%)	1/58 (1.7%)	**< 0.001**
knowledge of the violated content of acquaintances	115/170 (67.6%)	42/58 (72.4%)	0.5

^a^Lower educational level: primary and secondary school diploma, none

^b^Not Aware: a parent who does not know that (a) there are no laws regulating the exposure of minors online; (b) online contents can be violated by other subjects; (c) online contents could remain accessible virtually forever; (d) online contents can be seen and stolen by “non-followers”

**Table 2:** Analysis of socio-cultural characteristics between parents who use online gaming apps (APP Yes) and parents who do not use gaming apps (APP No) to entertain their children

Variables	APP Yes	APP No	*P* value
Full-time employment	42/91 (46.2%)	79/137 (57.7%)	0.08
Lower educational status^a^	15/91 (16.5%)	8/137 (5.8%)	**0.009**
Not aware^b^	89/91 (98%)	124/137 (90.5%)	**0.03**
Single parent	15/91 (16.5%)	19/137 (14%)	0.5
Compiler age < 35y	23/91 (25.3%)	47/137 (34.3%)	0.1
Child age > 8y	49/91 (53.8%)	40/137 (29.2%)	**< 0.001**

^a^Lower educational level: primary and secondary school diploma, none

^b^Not Aware: a parent who does not know that (a) there are no laws regulating the exposure of minors online; (b) online contents can be violated by other subjects; (c) online contents could remain accessible virtually forever; (d) online contents can be seen and stolen by “non-followers”

### Population

#### Inclusion criteria

Parents who had children between zero and 17 years old, who accessed the general outpatient clinic of Pediatrics at Umberto I Teaching Hospital were enrolled in the study. Employees of the Umberto I Teaching Hospital and/or of the Sapienza University of Rome, Italy who had children between 0 and 17 years old were also included. Parents had to sign the informed consent and then complete the whole questionnaire.

#### Exclusion criteria

We excluded parents whose children were older than 17, parents who did not agree to participate by signing the informed consent and those who did not want to complete the questionnaire.

### Statistics

According to a French study “Parents influenceurs” published by the “Observatoire de la Parentalité & de l’Éducation numérique”, 40% of parents in Western societies publish photos or videos of their children on social platforms [8]. The sample size was calculated based on the aforementioned study and the number of parents to be enrolled, 369, was processed on Calculator.net. The variables entered were the following: 95% confidence level, 5% confidence interval, 40% population.

The results obtained from the anonymous questionnaire were first analyzed by the automatic statistical counting of Google forms, then, qualitative variables were created on Excel sheets, and a second statistical analysis was carried out through the SPSS IBM Statistics program, using the parametric chi square test. Results with a *p* value < 0.05 were considered significant.

## Results

**Fig. 1:**
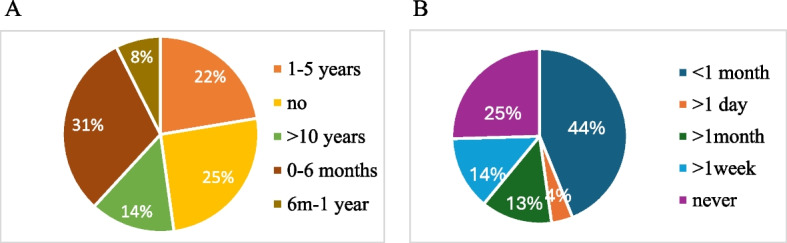
Pie charts describing age of the child when parents started sharing related contents online and frequency. **A** Percentages related to the number of parents answering the question about how old their child was when they stared sharenting. **B** Percentages related to the number of parents answering the question about how frequent they post contents related to their child online

**Fig. 2:**
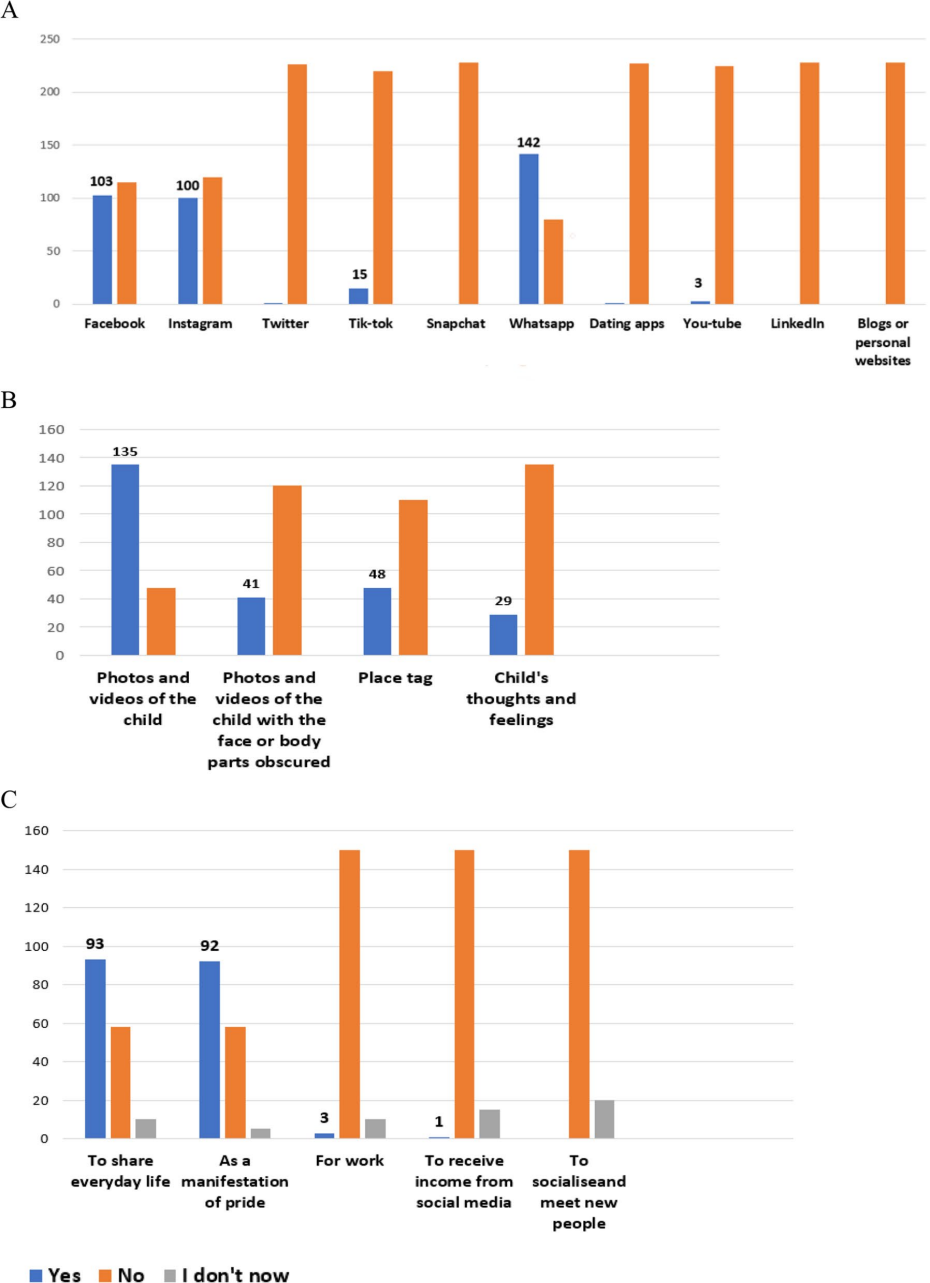
Bar graphs describing social online platforms used for sharing, content types, and reasons behind the sharing. **A** Bar graphs represent the number of parents using different online platforms for sharenting (the most used social platforms for sharing content (photos and/or videos) about children are Whatsapp (63.3%), Facebook (46%) and Instagram (44.6%)). **B** Bar graphs representing the different posted contents (the most posted content are photos and/or videos of the child (74.4%), followed by photos and/or videos with parts of the body and face obscured (24%); thoughts and feelings are shared in 17% of cases. Location tagging is used in 28.2% of postings). **C** Bar graphs representing the reasons why parents share their children related contents online (41% of parents answered that they posted to “share everyday life”, 40% as a manifestation of pride, while only 3 (3%) subjects admitted to do it for work and 1 receives income from this activity by social media)

A total of 228 parents of children under 18 years of age were surveyed (82% mothers, 18% fathers). Of them, 31% were under 35 years of age and 69% were over 35 years of age, and 98% were European. Ninety-eight percent of the respondents used social media. Among parents using one or more social media, 75% posted content about their children online; 25% of them stated that they have never posted content about their children on social media. We found that, compared to parents who do not post photos of their children (Gnp), parents who post photos of their children (Gp), tended to have part-time employment (29.3% vs. 53%, *p* = 0.002), a lower level of education (3.4% vs 12.4%, *p* = 0.05), an age younger than 35 (17.2% vs 35.3%, *p* = 0.01), and a number fi followers greater than 500 on social platforms (1.7% vs 21.2%, *p* =  < 0.001) (Table 1). We also found that the majority of parents who post children’s content (n 70, 31%) started within the first 6 months of life (Fig. 1A). A total of 100 interviewed (44%), state that postings occur less than once a month, while only 4% (n 9) state that they post content more than once a day (Fig. 1B). The most used social platforms for sharing content (photos and/or videos) about children are Whatsapp (63.3%), Facebook (46%) and Instagram (44.6%) (Fig. 2A).

The most posted content are photos and/or videos of the child (74.4%), followed by photos and/or videos with parts of the body and face obscured (24%); thoughts and feelings are shared in 17% of cases. Location tagging is used in 28.2% of postings (Fig. 2B).

When asked about the reasons why sharing information about their children on social platforms, 41% of parents answered that they posted to “share everyday life”, 40% as a manifestation of pride, while only 3 (3%) subjects admitted to do it for work and 1 receives income from this activity by social media (Fig. 2C). Generally, the interaction with the “followers”, expressed by the simple “like”, consequent to the posting of children’s photos and videos, generates satisfaction by the parent (82 interviewed (36%) stated that they feel “very satisfied”, 39 (17%) on average satisfied, 49 (21%) not very satisfied, and 58 (25%) not at all satisfied).

**Fig. 3:**
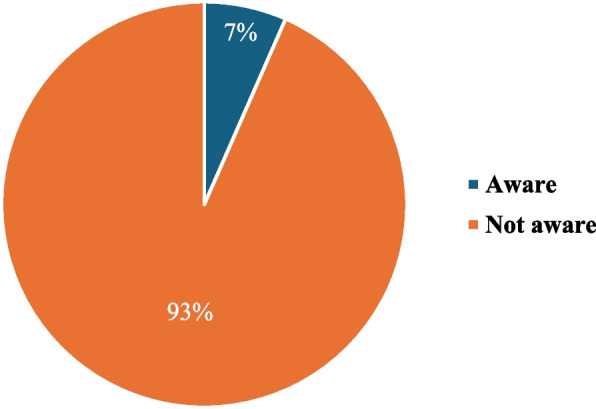
Awareness of “Sharenting” and related risks. Percentages related to the number of parents who are aware or not aware about the risks related to Sharenting. Not Aware: a parent who does not know that (1) there are no laws regulating the exposure of minors online; (2) online contents can be violated by other subjects; **c** online contents could remain accessible virtually forever; (3) online contents can be seen and stolen by ‘non-followers’. Aware a parent who knows all the above

We then investigated the parent’s level of awareness with respect to the issue of sharenting and the risks related to the exposure of minors on online platforms: we found that 93% of the respondents were not aware (Fig. 3).

Given the risk also associated with the use of online gaming app, we asked parents whether or not they let the child use online gaming apps, 40% admitted that habit. The analysis showed that gaming apps are used more by parents who have a lower level of education (46.2% vs 57.7%, *p* = 0.009), parents who are not aware of the risks related to the use of online platforms (98% vs 90.5%, *p* = 0.03), and, finally, parents with children older than 8 years of age (53.8% vs 29.2%, *p* < 0.001) (Table 2).

**Fig. 4:**
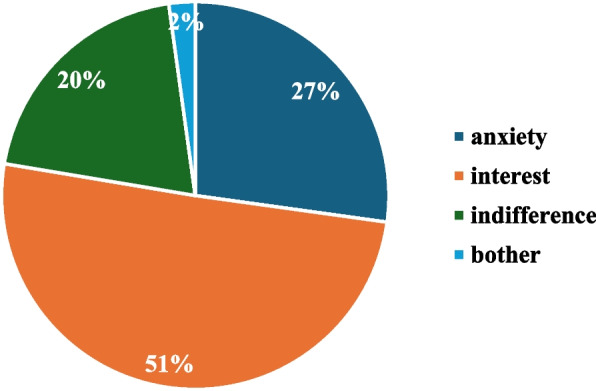
Parent’s feeling after completing the questionnaire about the topic Sharenting. Percentages of the compilers’ final reactions to the questionnaire

The questionnaire generated different reactions to the interviewed parents: 51% said they were interested and wanted to learn more about sharenting, 27% felt more anxious about the topic, 20% said they were indifferent, and 2% felt annoyed by the questions (Fig. 4).

## Discussion

In the phenomenon of “Sharenting”, the main focus is related to the privacy and online exposure of the child.

As highlighted in this study, sharing contents children-related is fueled by the number of followers parents have on their social channels (Table 1), this result is in line with what has been shown in other studies [9, 10]. Generally, the high number of followers correlates with greater activity on social media platforms, with more photos and videos posted, as such practice generates interactions which is linked to a sentiment of “satisfaction” and in some cases even material incomes including gifts, sponsorships and money.

Our study also found that sharenting is more frequent among parents with age < 35 years and that content sharing begins in the first 6 months of the child’s life (Fig. 1A); these observations suggest that sharenting is more frequently practiced in the early stages of parenthood, when parents, especially mothers, are more likely to feel isolated and lonely, as also highlighted in other studies in the literature [11, 12]; however, our questionnaire did not investigate the psychological aspect of the phenomenon and further studies are necessary to confirm this hypothesis.

Among the reasons why parents post their children’s content, it emerged how the integration of social media into people’s lives has generated the need to share personal events with the virtual community (the first reason for sharing in our cohort of interviewed parents). In addition, the pride felt by the parent towards her/his child (Fig. 2C), resulted to be the prominent feeling of parents sharing their children’s content with the virtual community, in line with other published analysis of this phenomenon [13].

As also demonstrated in a recent study [11], parents under 35 feel the need to make their new parental status public and to prove that they are ‘good parents’; in a time when our lives are deeply influenced by the technology and real identities overlap with digital identities, this need is satisfied by sharing of their children’s lives and habits on social networks; real social fulfilment therefore seems to necessarily pass through virtual social platforms.

An alarming data emerged from our analysis is the lack of awareness among parents regarding the status of sharenting related legislation, the risks and the privacy policies of social media platforms. The continuous online exposure of children entails several risks, including the violation of privacy and confidentiality, which is a children’s right, as enshrined in the Convention on the Rights of the Child and Adolescent and more recently in the General Data Protection Regulation (GDPR).

Parents are considered the ‘custodians’ or ‘owners’ of their children’s personal data, including the use of such data [14]; however, the psychological repercussions in children when they discover later in life that they have been exposed on the online network without their explicit consent and knowledge can be severe. A recent survey published in the Journal of Pediatrics and Child Health found that children aged between 4 and 17, surveyed through the administration of an age-adapted questionnaire, expressed the wish to be questioned and listened to before their parents shared stories or images about them on social media [15]. Finally, the most dramatic consequences of an uninformed use of children in social media are the risk of spreading content that can be used as material on pedophilia networks and the risk of luring through the use of online gaming apps [3, 6].

Our preliminary results show that parents who used online gaming apps to entertain their children have a lower educational level, are not aware of the risks and have children older than 8 years; the latter result suggests that this practice is used when the child is old enough to play independently. In light of these alarming statements and of the increasing use of social media, measures need to be taken to protect children from the ‘sharenting syndrome’ and the used of gaming apps.

Our study has some limitations. First, the sample size of the study is limited, and the majority of the compilers are parents of children attending the outpatients while few were employees of University Hospital, however this is a pilot study, and more data will be integrated in the final work. Our questionnaire was based on previously published surveys; however, it lacks validity measures. Finally, in order not to prolong the time needed to complete the questionnaire, we realized a survey lasting approximately 3 min, thus some information which could be useful, such as size of the family, and the distance between the children, are lacking.

## Conclusions

The results of our study could draw the attention of pediatricians, healthcare assistants and preventive healthcare professionals to the increasing practice of parents and families of publishing sensitive content about their children on online platforms and to the use of gaming apps among children and to the serious related risks. Pediatricians, healthcare assistants and prevention professionals should play a central role in informing families about the consequences of sharing child content on social media to increase awareness among parents.

## Data Availability

The data are available upon reasonable request and with permission of Lazio region. Requests to access should be directed to corresponding author.
